# BDNF val66met Polymorphism Impairs Hippocampal Long-Term Depression by Down-Regulation of 5-HT3 Receptors

**DOI:** 10.3389/fncel.2017.00306

**Published:** 2017-10-12

**Authors:** Rui Hao, Yu Qi, Dong-Ni Hou, Yuan-Yuan Ji, Chun-Yan Zheng, Chu-Yu Li, Wing-Ho Yung, Bai Lu, Ying Huang

**Affiliations:** ^1^Laboratory of Neuronal Circuit & Neuroplasticity, Department of Neurology, Tongji Hospital, Shanghai, China; ^2^Department of Physiology and Pharmacology, Tongji University School of Medicine, Shanghai, China; ^3^Department of Physiology and Pathophysiology, Shanghai Medical College, Fudan University, Shanghai, China; ^4^Neurodegeneration Discovery Performance Unit, GlaxoSmithKline (China) R&D, Shanghai, China; ^5^School of Biomedical Sciences, Faculty of Medicine, The Chinese University of Hong Kong, Shatin, Hong Kong; ^6^School of Pharmaceutical Sciences, Tsinghua University, Beijing, China

**Keywords:** BDNF val66met polymorphism, 5-HT3 receptors, LTD, hippocampus, synaptic plasticity

## Abstract

Brain-derived neurotrophic factor (BDNF) is a key regulator of neuronal plasticity and cognitive functions. BDNF val66met polymorphism, a human single-nucleotide polymorphism (SNP) in the pro-domain of BDNF gene, is associated with deficits in activity-dependent BDNF secretion and hippocampus-dependent memory. However, the underlying mechanism remains unclear. Here we show that in the BDNF^Met/Met^ mouse line mimicking the human SNP, BDNF expression in the hippocampus was decreased. There was a reduction in the total number of cells in hippocampal CA1 region, while hippocampal expression of mRNAs for NR2a, 2b, GluR1, 2 and GABA_A_Rβ3 subunits were up-regulated. Although basal glutamatergic neurotransmission was unaltered, hippocampal long-term depression (LTD) induced by low-frequency stimulation was impaired, which was partially rescued by exogenous application of BDNF. Interestingly, 5-HT3a receptors were down-regulated in the hippocampus of BDNF^Met/Met^ mice, whereas 5-HT2c receptors were up-regulated. Moreover, impaired LTD in BDNF^Met/Met^ mice was reversed by 5-HT3aR agonist. Thus, these observations indicate that BDNF val66met polymorphism changes hippocampal synaptic plasticity via down-regulation of 5-HT3a receptors, which may underlie cognition dysfunction of Met allele carriers.

## Introduction

Brain-derived neurotrophic factor (BDNF), a member of nerve growth factor family, plays an important role in the development and function of the central nervous system (CNS; Huang and Reichardt, 2001). In addition, it has been widely accepted that the main function of BDNF in the brain is to regulate synaptic plasticity. Exogenous application of BDNF facilitates theta burst stimulation (TBS) induced LTP in developing hippocampal slices (Figurov et al., 1996). Moreover, hippocampal LTP is impaired in heterozygous or homozygous BDNF-KO mice, and this impairment could be rescued by incubation with recombinant BDNF (Patterson et al., 1996). In the mammalian brain, BDNF is synthesized as the precursor of BDNF (proBDNF), which is then cleaved into mature BDNF by the tPA/plasmin system (Pang et al., 2004). A common single nucleotide polymorphism (SNP) rs6265 at nucleotide 196 (G/A) at codon 66 in the pro-domain of human BDNF gene converts the amino acid valine (Val) to methionine (Met). This amino acid substitution affects dendritic trafficking of pro-BDNF and alters the regulated secretion of BDNF (Egan et al., 2003; Chen et al., 2006). The allele frequency varies from 0.55% to 43.6% in Africa, Europe and Asia (Petryshen et al., 2010). Human subjects with this SNP exhibit deficits in short-term episodic memory, suggesting the importance of activity-dependent BDNF secretion. Growing evidence has implicated that Met allele carriers might have a higher susceptibility to Alzheimer’s disease and multiple psychiatric disorders (Bekinschtein et al., 2014; Lim et al., 2014; Notaras et al., 2015). In animal studies, it has been shown that the val66Met polymorphism decreases neurotransmission and impairs NMDAR-dependent synaptic plasticity in the hippocampus (Ninan et al., 2010). However, in dorsal striatum, the polymorphism enhances glutamatergic transmission but impairs synaptic plasticity (Jing et al., 2017). Also it has been revealed that in the IL-mPFC pyramidal neurons of BDNF^Met/Met^ mice, spike-timing-dependent plasticity (STDP) is absent, and NMDA receptor-mediated synaptic transmission is impaired (Pattwell et al., 2012).

Accumulating evidence suggests that serotonin (5-hydroxytryptamine, 5-HT) and its receptors are involved in many physiological or pathophysiological processes of cognitive function (Strüder and Weicker, 2001), as well as regulating synaptic plasticity. It has been revealed that 5-HT6 receptors are down-regulated in the cortical areas of patients with Alzheimer’s disease, correlating with the progression of disease and memory impairment (Garcia-Alloza et al., 2004). Iontophoresis of 5-HT2a receptor antagonists attenuates the memory fields of putative pyramidal cells in monkeys, whereas activation of 5-HT2a receptors accentuates the spatial tuning of these neurons (Williams et al., 2002). Administration of 5-HT3 receptors agonist m-CPBG at low and high doses impairs short-term or long-term memory (Meneses, 2007). Moreover, application of 5-HT inhibits hippocampal long-term depression (LTD) and the effect could be blocked by 5-HT1a receptor antagonist (Normann and Clark, 2005). *In vivo* study, it has been found that 5-HT4 receptor agonist blocks learning-induced depotentiation of LTP; whereas 5-HT4 antagonist enhances the intermediate phase of LTD and converts short-term depression into persistent LTD (Kemp and Manahan-Vaughan, 2005).

It is notable that high level of BDNF and 5-HT receptors are both expressed in the hippocampus (Wetmore et al., 1994; Barnes and Sharp, 1999). Growing evidence suggests a complex interaction between BDNF and 5-HT system. For example, expression of 5-HT receptors is altered in BDNF^+/−^ mice, correlating with the enhanced aggressiveness and hyperphagia (Lyons et al., 1999). Application of exogenous BDNF results in down-regulation of 5-HT2a receptors (Trajkovska et al., 2009), while BDNF expression is increased by a deficiency of 5-HT2c receptors, (Hill et al., 2011). There is good evidence that antidepressants including selective serotonin reuptake inhibitors (SSRIs) enhance the expression of BDNF mRNA (Castrén, 2004). However, the precise impact of val66met polymorphism on hippocampal synaptic plasticity and its underlying mechanism about the cross-talk with 5-HT system have not been well understood. In this study, using BDNF Met knock-in mice (BDNF^Met/Met^), we examined the effect of BDNF val66Met mutation on synaptic plasticity in CA1 region in the hippocampus and its mechanisms. We found that BDNF val66met polymorphism attenuates hippocampal LTD via the down-regulation of 5-HT3a receptors.

## Materials and Methods

### Animals

BDNF val66met polymorphism mouse strain was used in this study. All animals were on C57BL/6 background and male BDNF^Met/Met^ and BDNF^Val/Val^ mice derived from heterozygous BDNF^Val/Met^ parents were used. The details of the targeting construct and homologous recombination were described previously (Chen et al., 2006). All animal experiments were performed with protocols approved by the Animal Ethics Committee of Tongji University. The genotypes of animals were analyzed by preparing tail DNAs and PCR assay.

### Slice Preparation

Six to eight weeks (for LTP recording) or 3 week-old (for LTD recording) WT and BDNF^Met/Met^ mice were anesthetized by isoflurane and decapitated. The brains were rapidly dissected and placed in ice-cold artificial cerebrospinal fluid (ACSF, saturated with 95% O_2_ and 5% CO_2_), containing (in mM): NaCl (119), NaHCO_3_ (26.2), NaH_2_PO_4_ (1), KCl (2.5), CaCl_2_ (2.5), MgSO_4_ (1.3) and D-glucose (11). Subsequently, coronal hippocampal slices (400 μm) were prepared in oxygenated ACSF using a Leica VT1000S vibratome (Leica Instruments) at 4–6°C and maintained in ACSF at 25°C for at least 1 h before use.

### Electrophysiological Recording

The slices were submerged in the recording chamber, and perfused with ACSF containing (in mM): NaCl (119), NaHCO_3_ (26.2), NaH_2_PO_4_ (1), KCl (2.5), CaCl_2_ (2.5), MgSO_4_ (2.5) and D-glucose (11) at a rate of 2–4 ml/min at 25°C. Besides, BDNF were perfused at a concentration of 40 ng/ml in 1% BSA; 5-HTR3a agonist m-CPBG was perfused at a concentration of 1 μmol/L; 5-HT2cR antagonist ketanserin was perfused at a concentration of 20 μmol/L. Brain slices were perfused with chemicals for at least 30 min prior to the induction of LTP and LTD.

All electrophysiological recordings were performed with an Axopatch-200B amplifier (Molecular Devices, Sunnyvale, CA, USA) at the sampling rate of 10 kHz and filtered at 5 kHz. Field excitatory postsynaptic potentials (EPSPs) were recorded using 1.5–3.5 MΩ glass pipettes filled with ACSF and placed in the stratum radiatum of the CA1 region. Data were acquired and subsequently analyzed using ClampFit 9.0 software (Molecular Devices, Sunnyvale, CA, USA). A bipolar stimulating electrode was positioned at the terminals of the Schaffer collaterals (SC). Input/output relation of EPSP was assessed by electrical stimulation with intensities ranging from 20 pA to 140 pA. Stimulus were delivered every 30 s and repeated for five times at each intensity. Paired-pulse responses were evoked at inter-stimulus intervals of 60, 80, 100, 200, 300, 400 and 500 ms using a stimulation intensity of 0.5 mA. The paired-pulse ratio is defined as the ratio of second population spike amplitude to the first population spike amplitude. For LTP and LTD experiments, electrical stimulation was delivered every 30 s in recordings, consisting of low-intensity, square-wave pulses (0.1 ms). Theta-burst LTP was induced by three bursts (each burst having 4 pulses at 100 Hz) delivered at 5 Hz TBS. NMDAR-dependent LTD was induced by 900 pulses at 1-Hz stimulation (low frequency stimulation, LFS). Baseline responses were recorded for 15 min prior to stimulation. Responses were subsequently recorded for an additional 60 min to monitor changes in synaptic transmission. The synaptic strength was determined by the slope from 10% to 90% of the rising phase of the field EPSP (fEPSP). The magnitude of LTP or LTD was quantified as the normalized average slope of the fEPSP taken from the last 15 min of recording.

### Quantigene Assay

Hippocampus of 3 or 6-week-old mice were mechanically homogenized with the homogenization buffer of Quantigene 2.0 assay kit (Affymetrix, Santa Clara, CA, USA), which contains 1% protease Kinase K, followed by incubation at 65°C for 3 h with 800 rpm shaking in the Eppendorf incubator. Supernatants were collected after centrifugation at 21,200 *g* for 10 min. Quantigene probe sets for BDNF and GAPDH were customized from Panomics/Affymetrix.

The detection was performed following manufacture’s instruction of kit. Generally, after recovering the plates and reagents at RT, 60 μl of working probe sets were added to the plate and 40 μl of prepared tissue lysates were dispended into each well. Following incubation at 55°C overnight and a three-times wash step, the plates were added with 100 μl pre-amplifier working regents and incubated for 1 h at 55°C. Hybridized the 2.0 amplifier by adding 100 μl amplifier working reagent into each well after the wash step. Sealed the plates and incubated at 55°C for 1 h. After wash, the plates were further incubated with 100 μl 2.0 probe working reagent at 50°C for 1 h followed by complete wash steps. Signals were detected by Luminometer within 5–10 min after adding of 100 μl substrate into the wells. Signal of BDNF was normalized to signal of GAPDH in each sample tested.

### ELISA

Hippocampal tissues of 3 or 6-week-old mice were homogenized mechanically with lysis buffer at the ratio of 1:10 (w/v), followed by sonication for 10 s. Then placed on ice for 30 min. Supernatants were collected after centrifugation at 21,200 *g* for 15 min. Five microliter of supernatants from each sample were added for BDNF Elisa with another 1 μl used for protein concentration measurement (BCA assay).

BDNF ELISA was performed following manufacture’s instruction provided in kits (Human BDNF Quantikine SixPak, R&D, Minneapolis, MN, USA). Generally, after recovering the plates and reagents at RT, 100 μl standard assay diluents were added to each well. Fifty microliter BDNF standards prepared in RD5K or 5 μl sample plus 45 μl RD5K was added. After 2 h incubation at 600 rpm shaking, washed the plate for three times. One-hundred microliter BDNF conjugates were added to each well followed by 1 h incubation at 600 rpm shaking. Plate was washed for five times. Colors were developed by adding 200 μl mixture of reagent A&B and stopped by 50 μl stop solution. Mixed to make a homogenous yellow color. Absorbance was detected by Biotek plate reader at 450 nM with background set at 540 nM. The BDNF level in each sample were calculated against the standard curve and normalized to respective total protein concentration.

### Realtime-PCR

Hippocampal tissues were collected from 6-week-old BDNF^Met/Met^ and BDNF^Val/Val^ mice. mRNA was extracted using Trizol Reagent (Invitrogen, Carlsbad, CA, USA) according to manufacturer’s instruction. cDNA was synthesis with ReverTra Ace qPCR RT Kit (Toyobo, Japan). The expression of target genes were further analyzed with SYBR^®^Green Realtime PCR Master Mix (Toyobo, Japan) using StepOnePlus^™^ Real-Time PCR System (Applied Biosystems, Foster City, CA, USA). The following primers were used in qRT-PCR:

*GAPDH* (F: 5′-GAAGCCCATCACCATCTT-3′; R: 5′-CAGTAGACTCCACGACATAC-3′)
*NR1* (F: 5′-AAGGAGTGGAACGGAATG-3′; R: 5′-CTTGAAGGGCTTGGAGAA-3′)
*NR2a* (F: 5′-GATGACCAACGCTTAGTTATTG-3′; R: 5′-CT CAAGGATGACCGAAGATAG-3′)
*NR2b* (F: 5′-GGCCCTTGTCACCAATAA-3′; R: 5′-GTTGGACTGGTTCCCTATAC-3′)
*GluR1* (F: 5′-CTACGAGATCTGGATGTGTATAG-3′; R: 5′-CTTCACTGTGCCATTCATAAG-3′)
*GluR2* (F: 5′-TGACTGCGAAAGGGATAAA-3′; R: 5′-GCAGGTCTCCATCAGTAAA-3′)
*GABA-ARβ2* (F: 5′-CTTATCCCAGATTGTCCCTAA-3′; R: 5′-CCCAGGAGAGAATGGTAATC-3′)
*GABA-ARβ3* (F: 5′-ACAACTCAGGAATCCAGTATAG-3′; R: 5′-GTAGGTGGGTCTTCTTGTG-3′)
*GAD65* (F: 5′-TCAGCTCTCCTGGTTAGA-3′; R: 5′-GGACAGGTCATAGTGCTTAT-3′)
*GAD67* (F: 5′-TCTGGCTGATGTGGAAAG-3′; R: 5′-ATCTTGGCGTAGAGGTAATC-3′)
*5-HT1aR* (F: 5′-ATGGTGTCAGTGCTGGTGCT-3′; R: 5′- AGGTGCAGGATGGACGAAGT-3′)
*5-HT1bR* (F: 5′-CTGGTGTGGGTCTTCTCCAT-3′; R: 5′-GA CCGTGTAGAGGACGTGGT-3′)
*5-HT2cR* (F: 5′-AGAGGCACCATGCAAGCTAT-3′; R: 5′-AAGCACCGACAGGATATTGG-3′)
*5-HT3aR* (F: 5′-CTGTTGGCCTTGTTCCTTTC-3′; R: 5′-GT TAGCCAGGAGGTGGTCTG-3′)
*5-HT4R* (F: 5′-GCTGAGATGGTTCGTGTCAA-3′; R: 5′-GA TCCCGCTACAACGTCAGT-3′)
*5-HT6R* (F: 5′-AGCTCAGGCCGTATGTGACT-3′; R: 5′-CT CTTGAAGTCCCGCATGA-3′)
*5-HT7R* (F: 5′-TCTCGGTGTGCTTTGTCAAG-3′; R: 5′-TGAGGTCCGTGACACTAACG-3′)

Levels of mRNA expression were calculated by the ∆∆Ct method and normalized against *GAPDH* expression level.

### Western Blot

Corresponding hippocampal tissues as used for qRT-PCR analysis were collected and homogenized. Concentration of protein was determined using BCA assay. Thirty microgram of protein were loaded for electrophoretic separation (SDS-PAGE) and transferred on polyvinylidene difluoride (PVDF) membrane. Membranes were subsequently incubated with slim milk at room temperature for 1 h. Primary antibody against 5-HT2cR, 5-HT3aR, 5-HT4R (5-HT2cR: rabbit polyclonal to 5-HT2c receptor, 1:2000, Abcam, USA; 5-HT3aR: rabbit polyclonal to 5-HT3a receptor, 1:2000, Abcam, USA; 5-HT4R: rabbit polyclonal to 5-HT4 receptor, 1:2000, Abcam, USA) were added to the membrane and incubated at 4°C overnight. Membranes were incubated with secondary antibody (horseradish peroxidase-labeled Goat Anti-Rabbit IgG, 1:1000, Beyotime, China) or mouse monoclonal antibody to β-actin (1:2000, Beyotime, China) for 2 h at room temperature after wash. Blots were revealed by developing reagent (Beyotime, China) and photographed by chemiluminescence imaging system (GE Healthcare, Chicago, IL, USA). The amount of 5-HT2cR, 5-HT3aR, 5-HT4R protein was quantified with ImageJ software (Google, Mountain View, CA, USA) and normalized to the amount of β-actin.

## Results

### Down Regulation of BDNF and Decrease in Total Cell Number in BDNF^Met/Met^ Mice

**Figure 1:**
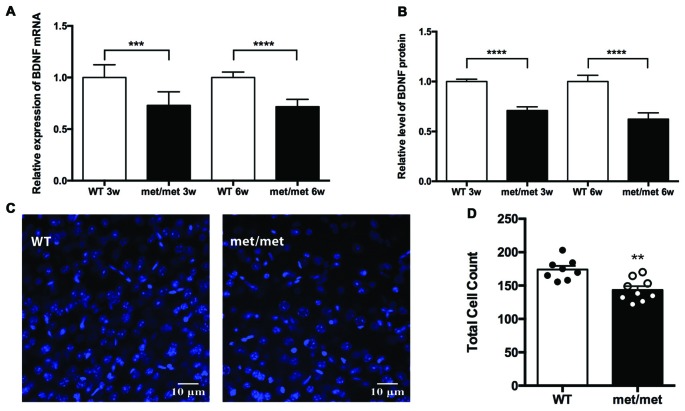
Expression of brain-derived neurotrophic factor (BDNF) and total cell number were down-regulated in BDNF^Met/Met^ mice. **(A)** Relative expression of BDNF mRNA by Quantigene assay (3w: WT: *N* = 8; BDNF^Met/Met^: *N* = 8; *P* < 0.001, 6w: WT: *N* = 8; BDNF^Met/Met^: *N* = 7; *P* < 0.0001). **(B)** Relative expression of BDNF protein by ELISA (3w: WT: *N* = 8; BDNF^Met/Met^: *N* = 8; *P* < 0.0001, 6w: WT: *N* = 8; BDNF^Met/Met^: *N* = 7; *P* < 0.0001). **(C)** Image of cells in the hippocampal CA1 region (6w). **(D)** Total cell count decreased in BDNF^Met/Met^ mice (*N* = 8 from three mice for WT; *N* = 9 from three mice for BDNF^Met/Met^). **P < 0.01; ***P < 0.001; ****P < 0.0001.

In the CNS including hippocampus, BDNF is secreted both constitutively and in a regulated manner in response to neuronal activity (Farhadi et al., 2000). It has been previously reported that val66met polymorphism selectively attenuated activity-dependent BDNF secretion, suggesting a possible underlying mechanism for impaired hippocampal function (Egan et al., 2003). To investigate the impact of BDNF val66Met polymorphism on the expression of BDNF in the hippocampus, we compared the levels of BDNF mRNA and protein between the WT and mutant mice. Using Quantigene assay, we found that expression of BDNF mRNA was down-regulated in the hippocampus of BDNF^Met/Met^ mice (3 or 6-week-old, Figure 1A; *P* < 0.0001 for Quantigene assay; unpaired *t*-test). Moreover, ELISA analysis showed that BDNF protein was decreased in the hippocampus of BDNF^Met/Met^ mice as well (Figure 1B, *P* < 0.0001, unpaired *t*-test).

As BDNF plays a pivotal role in the development and neurogenesis of nervous system, we then examined whether decreased level of BDNF alters the number of neurons during development. In the CA1 region of hippocampus, the total cell count decreased by 17.58% in BDNF^Met/Met^ mice, suggesting the effect of BDNF val66met polymorphism on cell survival (Figures 1C,D).

### Change of Glutamatergic and GABAergic Receptor Subunits in BDNF^Met/Met^ Mice

**Figure 2:**
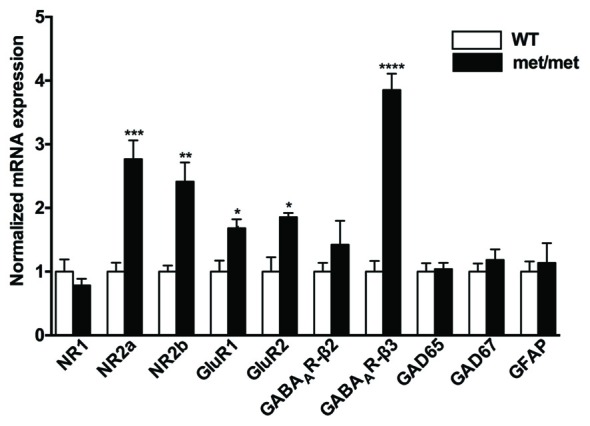
Altered mRNA expression of glutamatergic and GABAergic receptor subunits. NR2a and NR2b subunits for NMDA receptors, GluR1 and GluR2 subunits for AMPA receptors, as well as GABA_A_ receptor β3 subunit were up-regulated in BDNF^Met/Met^ mice (WT: *N* = 11; BDNF^Met/Met^: *N* = 10). *P < 0.05; **P < 0.01; ***P < 0.001; ****P < 0.0001.

BDNF has been implicated in regulating expression of glutamatergic and GABAergic receptors. Treatment of hippocampal neurons with BDNF regulates NR1, NR2a and NR2b NMDA receptor subunits (Caldeira et al., 2007) as well as GABA_A_ receptors (Brünig et al., 2001). To further elucidate the effect of BDNF val66Met polymorphism on NMDA, AMPA and GABA receptor subunits, we examined mRNA expression of NMDA receptor subunits (NR1, NR2a, NR2b), AMPA receptor subunits (GluR1, GluR2), GABA_A_ receptor subunits (β2 and β3), glutamic acid decarboxylases (GAD65 and GAD67) and GFAP (as a marker of glia cells) in hippocampus. As revealed by qPCR, we found that NR2a and NR2b subunits for NMDA receptors, GluR1 and GluR2 for AMPA receptors, as well as GABA_A_ receptor β3 subunits were up-regulated in BDNF^Met/Met^ mice (Figure 2), suggesting structure changes of these receptors, which may underlie synaptic transmission and plasticity remodeling.

### LFS Induced LTD Was Impaired in BDNF^Met/Met^ Mice

**Figure 3:**
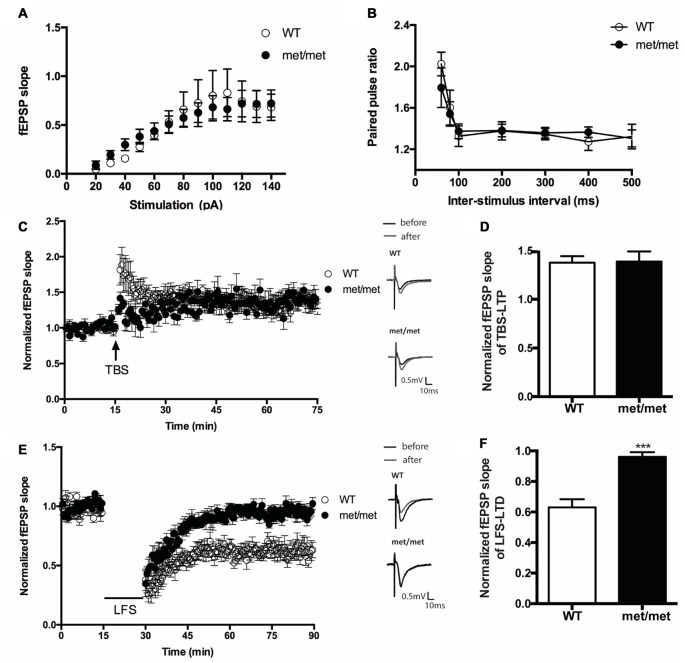
Hippocampal low frequency stimulation (LFS)-long-term depression (LTD), but not basal transmission and theta burst stimulation (TBS)-LTP, was impaired in BDNF^Met/Met^ mice. **(A)** Input-output curve (WT: *N* = 7 from four mice; BDNF^Met/Met^: *N* = 7 from four mice; *P* = 0.9758). **(B)** Pair-pulse ratio (WT: *N* = 6 from three mice; BDNF^Met/Met^: *N* = 10 from five mice; *P* = 0.8601). **(C)** TBS-induced CA3-CA1 LTP. Insert in **(C)**: typical field excitatory postsynaptic potential (fEPSP) recordings were shown before TBS (black) or 60 min after LTP induction (gray). **(D)** Summary of the data showed the magnitude of LTP. (*N* = 7 for WT; *N* = 5 for BDNF^Met/Met^). **(E)** LFS induced hippocampal LTD. Insert in **(E)**: typical fEPSP recordings were shown before LFS (black) or 60 min after LTD induction (gray). **(F)** Summary of the data showed the magnitude of LTD. (*N* = 7 for WT; *N* = 6 for BDNF^Met/Met^). ***P < 0.001.

To investigate the impact of BDNF val66met polymorphism on synaptic transmission, we examined the basal synaptic function of WT and BDNF^Met/Met^ mice. The input/output relation of fEPSP slopes and paired-pulsed ratio (for the intervals of 60, 80, 100, 200, 300, 400, 500 ms) in WT and BDNF^Met/Met^ mice were not different (Figures 3A,B), suggesting the basal transmission is not altered in mice with BDNF val66met polymorphism.

We also compared the stimulation induced LTP and LTD in slices from WT and BDNF^Met/Met^ mice. CA3- CA1 LTP was induced by SC TBS (TBS, three bursts, each having four pulses at 100 Hz, delivered at 5 Hz), whereas LTD was induced by low-frequency stimulation LFS (1 Hz, 900 pulses). We found that LTD was impaired in slices from BDNF^Met/Met^ mice (WT: 0.63 ± 0.053 of baseline, *N* = 7 from four mice; BDNF^Met/Met^: 0.96 ± 0.031 of baseline, *N* = 6 from four mice; *P* < 0.001, unpaired *t-test;* Figures 3E,F). However, the change of early-phase TBS-LTP was not detected in slices from BDNF^Met/Met^ mice during 1 h of recording (WT: 1.38 ± 0.066 of baseline, *N* = 7 from five mice; BDNF^Met/Met^: 1.39 ± 0.104 of baseline *N* = 5 from four mice; *P* > 0.05, unpaired *t* test; Figures 3C,D). These results suggest that the BDNF val66Met polymorphism attenuates LTD, but not early-phase LTP at the hippocampal CA3–CA1 synapses.

### Exogenous BDNF Partially Rescued LTD in BDNF^Met/Met^ Mice

**Figure 4:**
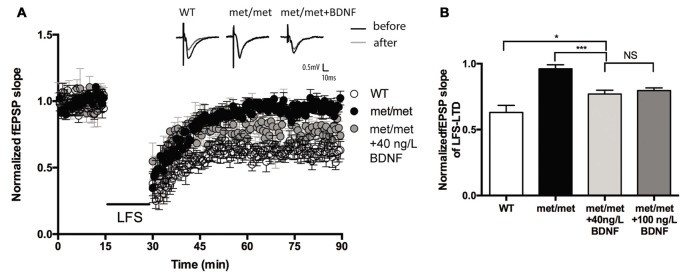
BDNF partially rescued LFS-LTD in BDNF^Met/Met^ mice. **(A)** LFS induced hippocampal LTD. Insert in **(A)**: typical fEPSP recordings were shown before LFS (black) or 60 min after LTD induction (gray). **(B)** Summary of the data showed the magnitude of LTD. (WT: *N* = 7 from four mice; BDNF^Met/Met^: *N* = 6 from four mice; BDNF^Met/Met^ +BDNF: 40 ng/ml, *N* = 7 from four mice; 100 ng/ml, *N* = 6 from four mice). *P < 0.05, ***P < 0.001.

Brain slices were then incubated with exogenous BDNF. BDNF (40 ng/ml, 30 min; 100 ng/ml, 60 min) was applied before LTD induction. Interestingly, the LTD deficit in slices of BDNF^Met/Met^ mice was partially rescued. The magnitude of LTD in BDNF^Met/Met^ slices was significantly increased after BDNF perfusion (BDNF 40 ng/ml, 30 min, WT: 0.63 ± 0.053 of baseline, *N* = 7 from four mice; BDNF^Met/Met^: 0.96 ± 0.031 of baseline, *N* = 6 from four mice; BDNF^Met/Met^ +BDNF: 0.77 ± 0.028 of baseline, *N* = 7 from four mice, *P* < 0.05, one-way ANOVA; Figure 4), suggesting that impaired LTD may be partially due to BDNF deficiency.

### Change of 5-HT Receptors Expression in BDNF^Met/Met^ Mice

**Figure 5:**
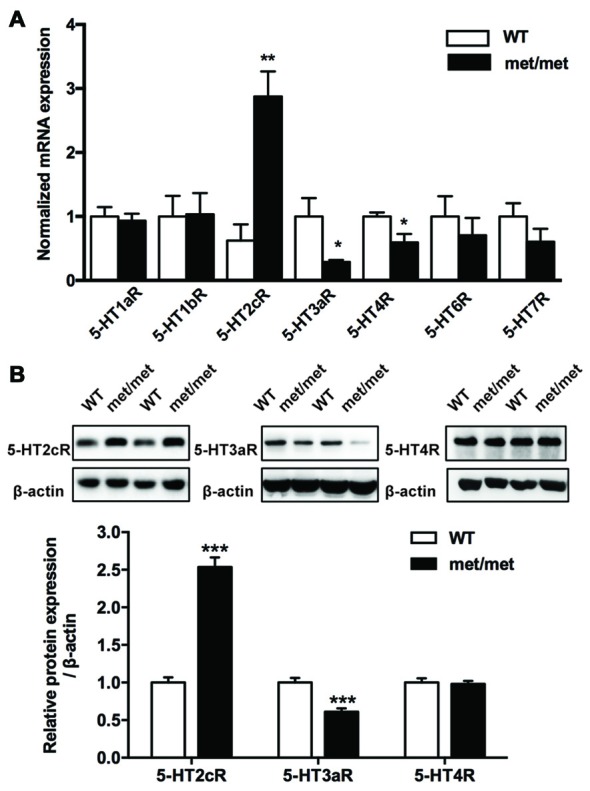
Altered expression of 5-hydroxytryptamine (5-HT) receptors. **(A)** qRT-PCR of 5-HT receptor gene in the hippocampus. The mRNA expression of 5-HT2cR was up-regulated, yet the mRNA of 5-HT3aR and 5-HT4R was down-regulated in the hippocampus of BDNF^Met/Met^ mice (WT: *N* = 11; BDNF^Met/Met^: *N* = 10). **(B)** Western blot analysis of the 5-HT receptor protein. 5-HT2cR protein increased, while 5-HT3aR protein decreased in BDNF^Met/Met^ mice (WT: *N* = 11; BDNF^Met/Met^: *N* = 10). *P < 0.05, **P < 0.01, ***P < 0.001.

The fact that BDNF could only partially rescue the LTP impairment indicates that secondary mechanisms other than BDNF deficiency maybe involved. Given the complex interaction between BDNF and 5-HT system (Lyons et al., 1999; Deltheil et al., 2008; Trajkovska et al., 2009), to investigate the potential impact of BDNF val66met polymorphism on the serotonin system, the levels of serotonin receptor mRNA in the hippocampus were analyzed by qPCR. We examined the expression of 5-HT1aR, 5-HT1bR, 5-HT2cR, 5-HT3aR, 5-HT4R, 5-HT6R and 5-HT7R, which have close relevance with memory and cognition (Seyedabadi et al., 2014). The mRNA expression of 5-HT2cR was up-regulated, while that of 5-HT3aR and 5-HT4R was down-regulated in the hippocampus of BDNF^Met/Met^ mice. Furthermore, the changes of 5-HT2cR and 5-HT3aR proteins were confirmed by western blot analysis. However, we failed to detect any diminishment in 5-HT4R protein (Figure 5). Thus, we further focused on the role of 5-HT2cR and 5-HT3aR in LTD impairment in BDNF^Met/Met^ mice.

### Impaired LTD in BDNF^Met/Met^ Mice Was Reversed by 5-HT3aR Agonist

**Figure 6:**
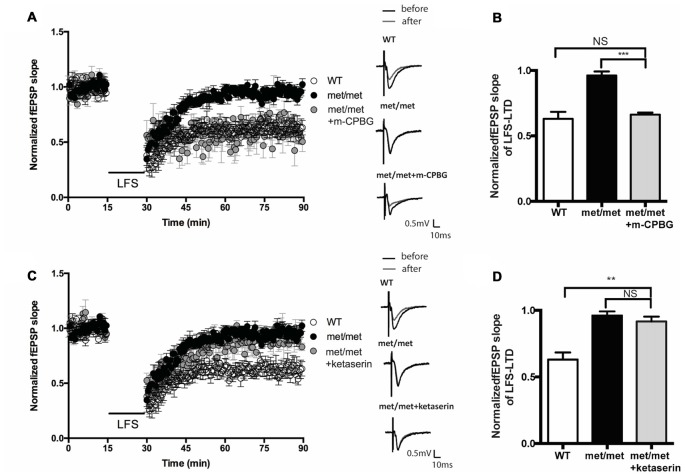
Effect of 5-HT2cR antagonist and 5-HT3aR agonist on LFS-LTD in BDNF^Met/Met^ mice. **(A)** LFS induced hippocampal LTD. Insert in **(A)**: typical fEPSP recordings were shown before LFS (black) or 60 min after LTD induction (gray). **(B)** Summary of the data showed 5-HT3aR agonist m-CPBG (1 μM) rescued LFS-LTD (WT: *N* = 7 from 5 mice; BDNF^Met/Met^: *N* = 6 from three mice; BDNF^Met/Met^ +m-CPBG: *N* = 7 from four mice). **(C)** LFS induced hippocampal LTD. Insert in **(C)**: typical fEPSP recordings were shown before LFS (black) or 60 min after LTD induction (gray). **(D)** Summary of the data showed 5-HT2cR antagonist ketanserin (20 μM) failed to rescue LFS-LTD (WT: *N* = 7 from four mice; BDNF^Met/Met^: *N* = 6 from four mice; BDNF^Met/Met^ + ketanserin: *N* = 6 from three mice). NS *P* > 0.05, **P < 0.01, ***P < 0.001.

BDNF^Met/Met^ slices were perfused with 5-HT3aR agonist m-CPBG (1 μM). The LTD deficit in BDNF^Met/Met^ slices was rescued to a similar level as that from WT slices (WT: 0.63 ± 0.053 of baseline, *N* = 7 from five mice; BDNF^Met/Met^: 0.96 ± 0.031 of baseline, *N* = 6 from three mice; BDNF^Met/Met^ + m-CPBG: 0.62 ± 0.054 of baseline, *N* = 7 from four mice, *P* < 0.05, one-way ANOVA; Figures 6A,B). Thus, the impairment of LTD in BDNF^Met/Met^ mice could be attenuated by 5-HT3aR agonist. In contrast, application of 5-HT2cR antagonist ketanserin (20 μM) failed to rescue the LTD deficit in BDNF^Met/Met^ slices (WT: 0.63 ± 0.053 of baseline, *N* = 7 from four mice; BDNF^Met/Met^: 0.96 ± 0.031 of baseline, *N* = 6 from four mice; BDNF^Met/Met^ + ketanserin: 0.92 ± 0.036 of baseline, *N* = 6 from three mice, *P* < 0.05, one-way ANOVA; Figures 6C,D). These results suggest that down-regulation of 5-HT3aRs is involved in LTD impairment by BDNF val66met polymorphism.

## Discussion

In the present study, using BDNF^Met/Met^ mice, we investigated the effect of BDNF val66met polymorphism on synaptic plasticity and the underlying mechanism. We demonstrated that BDNF expression and total cell number were down-regulated in the hippocampus in BDNF^Met/Met^ mice. Expression of mRNAs for NR2a, 2b, GluR1, 2 and GABA_A_Rβ3 subunits were up-regulated by BDNF val66met polymorphism. Moreover, hippocampal LTD was impaired, which was partially rescued by exogenous BDNF and totally reversed by 5-HT3aR agonist.

BDNF val66met polymorphism is a common SNP in the BDNF gene in human. Met-BDNF pro-peptide exhibits an altered trafficking and secretion of BDNF (Chen et al., 2004), leading to a decrease in activity-dependent BDNF release (Egan et al., 2003). A previous study showed that in the whole brain lysates of 21-day-old mice, the level of BDNF was similar in WT and BDNF^Met/Met^ mice; yet the number of BDNF-containing varicosities in BDNF-expressing cells was reduced in the hippocampus of BDNF^Met/Met^ mice (Chen et al., 2006). In this study, using multiple assays, we found that the expression of BDNF mRNA and protein was attenuated specifically in the hippocampus of 6–7 week-old BDNF^Met/Met^ mice. Thus, BDNF val66met polymorphism may selectively reduce BDNF levels in certain brain regions such as hippocampus. Given the crucial role of BDNF in neuronal survival during development, it is not surprising that the cell number in the hippocampus was attenuated as the consequence of BDNF deficiency.

It has also been reported that both early-phase and late-phase hippocampal LTP are impaired in 1-month-old BDNF^Met/Met^ mice, whereas in 4-month old mice, only late-phase LTP is reduced (Ninan et al., 2010), which indicates that early-phase LTP in adult animal is less sensitive to BDNF deficiency due to val66met polymorphism. It may be the possible reason for the unaltered early-phase CA3-CA1 LTP we found in BDNF^Met/Met^ mice (6–8 week-old) in this study. Moreover, earlier study revealed that LFS induced NMDAR-LTD, but not DHPG induced NMDAR-independent LTD was impaired in 1-month-old BDNF^Met/Met^ mice (Ninan et al., 2010). Here we found that LFS induced LTD was attenuated by BDNF val66met polymorphism consistently. Furthermore, we found that acute treatment with exogenous BDNF rescued the LTD deficit but incompletely, implying the possibility of additional mechanisms involved. BDNF is thought to have cooperative effect with 5-HT system. A deficiency of BDNF in BDNF^+/−^ mice results in the alteration of 5-HT receptor expression in lateral frontal cortex, hippocampus and hypothalamus (Lyons et al., 1999). Acute administration of BDNF in the hippocampus potentiates the uptake of 5-HT by activating TrkB (Deltheil et al., 2008). In this study, we found that hippocampal expression of 5-HT2cR was up-regulated, while 5-HT3aR was down-regulated in BDNF^Met/Met^ mice. Although our result of 5-HT2cR overexpression is different from the previous report that BDNF deficiency down-regulated hippocampal 5-HT2cR in 6–9 month-old mice (Lyons et al., 1999), which may be due to the different age of animals used in experiments, these results further confirmed the cross-talk between BDNF and 5-HT receptors.

In serotonin receptor family, 5-HT3 receptors (5-HT3Rs) are the only ionotropic receptor (Chameau and van Hooft, 2006; Faerber et al., 2007), which are specifically expressed in cholecystokinin (CCK)—containing inhibitory interneurons in the hippocampus and mediate fast synaptic transmission in the raphe-hippocampal interneuron synapses. The activity of 5-HT3Rs located on postsynaptic somatodendritic regions or presynaptic terminals modulates firing or neurotransmitter release of interneurons, which consequently regulates pyramidal synaptic plasticity via GABAergic projection from CCK interneurons and thus modulates memory (Rondé and Nichols, 1998; Sudweeks et al., 2002). Injection of the 5-HT3R antagonist granisetron into hippocampus impairs spatial learning (Naghdi and Harooni, 2005). Granisetron could also reduce new learning associated with extinction of fearful memories (Park and Williams, 2012). Our previous study found that 5-HT3aR disruption inhibited AMPA receptors internalization and impaired LTD (Yu et al., 2014). However, in contrast to 5-HT3Rs, 5-HT2 receptors are G-protein coupled receptors, which are also highly expressed in the hippocampus (Hoffman and Mezey, 1989) and modulate cognitive function. It has been revealed that 5-HTr2c antagonist facilitates long term memory although does not affect short term memory (Meneses, 2007). Moreover, spatial learning but not hippocampal LTP was impaired in 5-HTr2c mutant mice (Tecott et al., 1998). In this study, we found that acute application of exogenous 5-HT3aR agonist completely rescued the impaired LTD in BDNF^Met/Met^ mice, but 5-HT2cR antagonist did not reverse deficient LTD. These results suggest a role of the 5-HT3aR-dependent signaling in the LTD deficit in BDNF val66met polymorphism, whereas 5-HT2cR may not involve in this modulation. As ligand-gated ion channels, 5-HT3aRs have more rapid effect compared with other G-protein coupled receptors in serotonin receptor family, which may be more conducive to mediate or regulate fast synaptic transmission and plasticity. The down-regulation of 5-HT3aR in CCK interneurons may change GABA release at inhibitory synapses from these interneurons to pyramidal cells, altering their membrane depolarization. The disruption of depolarization-induced calcium influx and Ca^2+^-dependent phosphatase may contribute to impairments in AMPAR internalization and LTD. However, besides 5-HT3aRs, the impact of pro-BDNF deficiency on LTD could not be ruled out. In conclusion, this study revealed that BDNF val66met polymorphism impaired hippocampal LTD by reducing BDNF expression and down-regulation of 5-HT3aRs, and provided new insights into the modification of 5-HT receptors by BDNF val66met polymorphism and its contribution to the cognitive dysfunction of Met allele carriers.

## Author Contributions

YH and RH conceived and designed the study; RH, YQ, D-NH, C-YL, Y-YJ and C-YZ performed experiments; RH, YQ and C-YZ conducted data analysis; W-HY and BL assisted in experiments, data analysis; RH, YQ and YH wrote the manuscript; BL and W-HY contributed to the revisions.

## Conflict of Interest Statement

The authors declare that the research was conducted in the absence of any commercial or financial relationships that could be construed as a potential conflict of interest.
