# Corrigendum: BDNF val66met Polymorphism Impairs Hippocampal Long-Term Depression by Down-Regulation of 5-HT3 Receptors

**DOI:** 10.3389/fncel.2017.00351

**Published:** 2017-11-13

**Authors:** Rui Hao, Yu Qi, Dong-Ni Hou, Yuan-Yuan Ji, Chun-Yan Zheng, Chu-Yu Li, Wing-Ho Yung, Bai Lu, Ying Huang

**Affiliations:** ^1^Department of Neurology, Tongji Hospital, Tongji University School of Medicine, Shanghai, China; ^2^Department of Physiology and Pharmacology, Tongji University School of Medicine, Shanghai, China; ^3^Department of Physiology and Pathophysiology, Shanghai Medical College, Fudan University, Shanghai, China; ^4^Neurodegeneration Discovery Performance Unit, GlaxoSmithKline (China) R&D, Shanghai, China; ^5^School of Biomedical Sciences, Faculty of Medicine, The Chinese University of Hong Kong, Shatin, Hong Kong; ^6^School of Pharmaceutical Sciences, Tsinghua University, Beijing, China

**Keywords:** BDNF val66met polymorphism, 5-HT3 receptors, LTD, hippocampus, synaptic plasticity

In the original article, affiliation 1 was incorrect. The correct affiliation 1 should read “Department of Neurology, Tongji Hospital, Tongji University School of Medicine, Shanghai, China”.

Finally, we neglected to include the funder Beijing Municipal Science & Technology Commission, No. Z151100003915118 to Bai Lu. The corrected Funding section appears below.

The authors apologize for these errors and state that these do not change the scientific conclusions of the article in any way.

## Conflict of interest statement

The authors declare that the research was conducted in the absence of any commercial or financial relationships that could be construed as a potential conflict of interest.

